# Emergence of a unique group of necrotizing mycobacterial diseases.

**DOI:** 10.3201/eid0503.990307

**Published:** 1999

**Authors:** K M Dobos, F D Quinn, D A Ashford, C R Horsburgh, C H King

**Affiliations:** Emory University School of Medicine, Atlanta, Georgia 30303, USA.

## Abstract

Although most diseases due to pathogenic mycobacteria are caused by Mycobacterium tuberculosis, several other mycobacterial diseases-caused by M. ulcerans (Buruli ulcer), M. marinum, and M. haemophilum-have begun to emerge. We review the emergence of diseases caused by these three pathogens in the United States and around the world in the last decade. We examine the pathophysiologic similarities of the diseases (all three cause necrotizing skin lesions) and common reservoirs of infection (stagnant or slow-flowing water). Examination of the histologic and pathogenic characteristics of these mycobacteria suggests differences in the modes of transmission and pathogenesis, though no singular mechanism for either characteristic has been definitively described for any of these mycobacteria.


### Emergence of a Unique Group of Necrotizing Mycobacterial Diseases

**Karen M. Dobos,*† Frederick D. Quinn,† David A. Ashford,† C. Robert Horsburgh,* and C. Harold King***
*Emory University School of Medicine, Atlanta, Georgia, USA; †Centers for Disease Control and Prevention, Atlanta, Georgia, USA

Although most diseases due to pathogenic mycobacteria are caused by *Mycobacterium tuberculosis,* several other mycobacterial diseases—caused by *M. ulcerans* (Buruli ulcer), *M. marinum,* and *M. haemophilum*—have begun to emerge. We review the emergence of diseases caused by these three pathogens in the United States and around the world in the last decade. We examine the pathophysiologic similarities of the diseases (all three cause necrotizing skin lesions) and common reservoirs of infection (stagnant or slow-flowing water). Examination of the histologic and pathogenic characteristics of these mycobacteria suggests differences in the modes of transmission and pathogenesis, though no singular mechanism for either characteristic has been definitively described for any of these mycobacteria.

### Diseases Caused by Emergence of Atypical Mycobacteria

Mycobacterial diseases cause substantial illness and death throughout the world, despite years of public health control efforts. Although most illnesses and deaths are due to tuberculosis (1), particularly in developing countries and in association with the AIDS pandemic (2), diseases caused by nontuberculous mycobacteria (NTM) have had a strong impact on human populations in both developing and industrialized countries (3). Many NTM diseases, such as those caused by *Mycobacterium avium* complex, are considered opportunistic infections in patients with AIDS (4). However, the rates of non-AIDS-associated NTM infections are also increasing (5). Specifically, disease caused by *M. ulcerans*, *M. marinum*, and *M. haemophilum* has increased in both healthy and immunocompromised patients in the last decade. Moreover, these diseases have been reported from previously unaffected geographic areas, which indicates an increase in the geographic distribution of these organisms.

Of these three emerging NTM diseases, Buruli ulcer (BU), caused by *M. ulcerans*, poses the greatest immediate public health threat. Indeed, BU is rapidly becoming the third most prevalent mycobacterial disease, with an impact soon to surpass that of leprosy (6). Although it was first documented in Australia in 1947 (7), the disease was named after the Buruli District of Uganda (8) after an investigation of superficial, ulcerative lesions in Ugandan children. At the time, the disease was sporadically reported throughout Central and West Africa and Australia. In the past decade, incidence of this disease has dramatically increased, with cases now reported in most of sub-Saharan Africa, Mexico, Surinam, Peru, Bolivia, French Guiana, India, sporadically throughout southern Asia, and in Papua New Guinea (6,9). In addition, several cases have been reported in Belgium, Japan, Northern Ireland, and in the United States, resulting from international travel (6,10,11).

A retrospective investigation over a 10-year period in the Daloa region of Côte d'Ivoire was conducted to document increases in the incidence of BU. Cases increased dramatically over a 10-year period, with some villages demonstrating disease rates of 16% of the population at the end of the study (12). Current rates are estimated at more than 22% in some of these villages (6). Increases have also been reported in Australia, with outbreaks of BU during the 1990s in areas where the disease had

Address for correspondence: C. Harold King, Division of Infectious Diseases, Department of Medicine, Emory University School of Medicine, 69 Butler Street SE, Atlanta, GA 30303; fax: 404-880-9305; e-mail: cking01@emory.edu.

not been previously seen (13). These data most likely underestimate BU occurrence as there are no reliable tools for the surveillance and diagnosis of this disease other than clinical signs and symptoms.

Disease caused by *M. marinum* was observed in clusters of cases between 1930 and 1970, and *M. marinum* was well accepted as a human pathogen before the 1980s. In contrast, *M. haemophilum* was a rarely identified pathogen before 1974. A retrospective study conducted in 1974 reported that, on the basis of epidemiologic evidence, *M. haemophilum* was the causative agent of a syndrome that included mycobacterial adenitis and skin lesions that developed in 29 immunocompetent patients (14). In 1978, the bacterium was identified as the cause of cutaneous ulcerating lesions in a woman with underlying Hodgkin disease (15). Subsequently, *M. haemophilum* has been described as causing cutaneous lesions in persons receiving immunosuppressive therapy after a renal transplant (16).

The occurrence of *M. marinum* and *M. haemophilum* in human disease is likely underreported, as diagnosis of the diseases caused by *M. marinum* and *M. haemophilum* is frequently missed. Nonetheless, confirmed cases of these diseases have been increasing, both internationally (5) and within the United States. A national survey involving 46 state and local laboratory centers, representing 33 states and the District of Columbia, was conducted from 1981 to 1983 to determine the prevalence of NTM diseases. Fifty-three cases of NTM disease caused by *M. marinum* and one case caused by *M. haemophilum* were reported over the 3-year period (17), for a national average number of cases of 40 and 0.76 respectively, per year.

In 1993, a laboratory-based surveillance system (18) began to assess the recent prevalence of NTM disease in the United States. For the *Mycobacterium* module, the population under surveillance included patients in the United States who had a specimen submitted to the state laboratories for evaluation. It is not known what percentage of all mycobacterial isolates this represented. Only one isolate per person was recorded. The geographic distribution and number of cases of *M. marinum* disease (40 states reporting) and *M. haemophilum* disease (9 states reporting), submitted from 1993 to 1996, are presented in Tables 1 and 2, respectively.

Table 1. Laboratory-confirmed cases of *Mycobacterium marinum* in the five regions of the United States (40 states reporting), by year, 1993–1996[a]

| | No. cases (%) | | | |
|---|---|---|---|---|
| Region | 1993 | 1994 | 1995 | 1996 |
| Northeast | 21 (14) | 40 (22) | 34 (23) | 28 (18) |
| Southeast | 58 (38) | 66 (37) | 41 (28) | 64 (41) |
| North central | 43 (28) | 38 (21) | 27 (27) | 38 (24) |
| South central | 17 (11) | 17 (10) | 17 (17) | 14 (9) |
| Mountain | 8 (5) | 11 (6) | 13 (13) | 7 (5) |
| Pacific | 5 (3) | 7 (4) | 15 (15) | 6 (4) |
| Total | 152 | 179 | 147 | 157 |

[a]These data are reported as part of the passive laboratory-based surveillance system using the Public Health Laboratory Information System software developed by the Centers for Disease Control and Prevention and the Association of State and Territorial Public Health Laboratory Directors.

Table 2. Laboratory-confirmed cases of *Mycobacterium haemophilum* in the five regions of the United States (9 states reporting), by year, 1993–1996[a]

| | No. cases (%) | | | |
|---|---|---|---|---|
| Region | 1993 | 1994 | 1995 | 1996 |
| Northeast | 0 | 0 | 0 | 0 |
| Southeast | 11 (79) | 0 | 0 | 2 (50) |
| North central | 1 (7) | 0 | 0 | 0 |
| South central | 0 | 1 (25) | 0 | 0 |
| Mountain | 2 (14) | 3 (75) | 3 (100) | 2 (50) |
| Pacific | 0 | 0 | 0 | 0 |
| Total | 14 | 4 | 3 | 4 |

[a]These data are reported as part of the passive laboratory based surveillance system using the Public Health Laboratory Information System software developed by the Centers for Disease Control and Prevention and the Association of State and Territorial Public Health Laboratory Directors.

These data demonstrate that the number of cases of these two diseases in the United States has increased from the past decade, with the estimated national average number of cases of 198 and 35 per year, respectively. In addition, cases of *M. marinum* in several states over the 4-year period of this survey have increased (Table 3) (cases of *M. marinum* had not been reported in Missouri previously [17]). Elsewhere, increases in *M. marinum* disease have been reported throughout the world in temperate climates (5).

In the United States, most cases of *M. haemophilum* disease are still found in the South; however, *M. haemophilum* disease has been described in the New York City metropolitan area (19). In addition, the number

Table 3. Laboratory-confirmed cases of *Mycobacterium marinum* reported by individual states within the United States, by year, 1993–1996[a]

| State | No. cases (%)[b] 1993 | 1994 | 1995 | 1996 |
|---|---|---|---|---|
| Florida | 15 (9.9) | 13 (7.3) | 13 (8.8) | 24 (15.3) |
| Maryland | 15 (9.9) | 24 (13.4) | 21 (14.3) | 22 (14.0) |
| Minnesota | 6 (4.0) | 4 (2.2) | 6 (4.1) | 8 (5.1) |
| Missouri | 2 (1.3) | 7 (3.9) | 5 (3.4) | 9 (5.7) |
| Utah | 3 (1.9) | 2 (1.1) | 5 (3.4) | 4 (2.6) |
| Virginia | 7 (4.6) | 13 (7.3) | 12 (8.2) | 11 (7.0) |
| Wisconsin | 9 (5.9) | 8 (4.5) | 9 (6.1) | 9 (5.7) |

[a]These data are reported as part of the passive laboratory-based surveillance system using the Public Health Laboratory Information System software developed by the Centers for Disease Control and Prevention and the Association of State and Territorial Public Health Laboratory Directors.
[b]Percent (%) denotes contribution to cases reported nationally for the year.

of cases of *M. haemophilum* disease in the United States is expanding (Table 2). Thought to occur only in immunocompromised persons (with organ transplant patients and persons with AIDS representing most of the patients) (20), *M. haemophilum* disease was rarely reported even in these populations before 1990. By 1994, 40 cases of *M. haemophilum* disease associated with immunocompromised persons had been reported worldwide (20). However, more recently, a report by Saubolle and colleagues (19) described 10 cases of *M. haemophilum* disease in Arizona (1984 to 1994), which included cases in two otherwise healthy children and three in adults undergoing corticosteroid therapy for rheumatoid arthritis or Crohn disease. Additional cases of *M. haemophilum* disease in otherwise healthy children (21) and adults have recently been observed (M.A. Saubolle, pers. comm.). Elsewhere, *M. haemophilum* disease has been reported in Australia, Canada, France, Israel, and the United Kingdom (19).

### Clinical and Histologic Features

### Buruli Ulcer

*M. ulcerans* causes a skin disease commonly known as BU. The incubation period can be highly variable but is generally less than 3 months (22). The ulcers are indolent and necrotizing (9). Systemic signs and symptoms, such as fevers or weight loss, and bacterial superinfection are rare (12,22). Erythema and induration are present at the onset of infection but subside rapidly with the beginning of ulceration. Healing usually takes 4 to 6 months and involves extensive scar formation. This scarring frequently results in deformity, particularly in children, in whom the result can be joint contracture, subluxation, disuse atrophy, or distal lymphedema. Circumferential cicatrization may lead to stunted limb growth. In one series, 26% of patients were left with functional disability of a limb (12). However, death from BU is rare, and no disseminated disease has been reported in either healthy or immunosuppressed persons.

Histologically, *M. ulcerans* produces a circumscribed area of necrosis and (unlike most other mycobacterial pathogens) infected tissues that primarily contain extracellular bacilli, with microcolonies containing large numbers of extracellular acid-fast bacilli (AFB) in the center of the lesions and in association with adipose cells (Figure 1A, B). The effect of AFB at the site of infection can be extensive during the preulcerative phase, with few to no intracellular AFB present (Figure 1B). The lesions are symmetrical with associated coagulation necrosis of the deep dermis and panniculus. The lesions very rarely penetrate beyond the fascia to associate with the underlying muscle. Necrosis occurs extensively beyond the central regions with destruction of capillaries, larger vessels, and adipose cells (Figure 1A) (23). The AFB localize to the adipose tissue (Figure 1B) with necrosis of the adipose tissues occurring at sites distant to the location of the bacilli (Figure 1A) and extensive AFB in all preulcerative nodules and early lesions. The necrosis and damage of the dermis lead to ulceration of the overlying skin. As the ulceration spreads through the panniculus, hypersensitivity granulomas, most likely stimulated by mycobacterial antigens, develop in the dermis and other tissues surrounding the lesions.

### *M. marinum* Disease

*M. marinum* causes small ulcers or nodules, usually on the extremities (24). The incubation period is approximately 2 weeks to several months (24). These lesions are minimally painful and usually heal in 1 to 2 years without treatment. Main symptoms include slight tenderness and discharge from the necrotic sites. In fewer than 10% of cases, localized lymphangitic

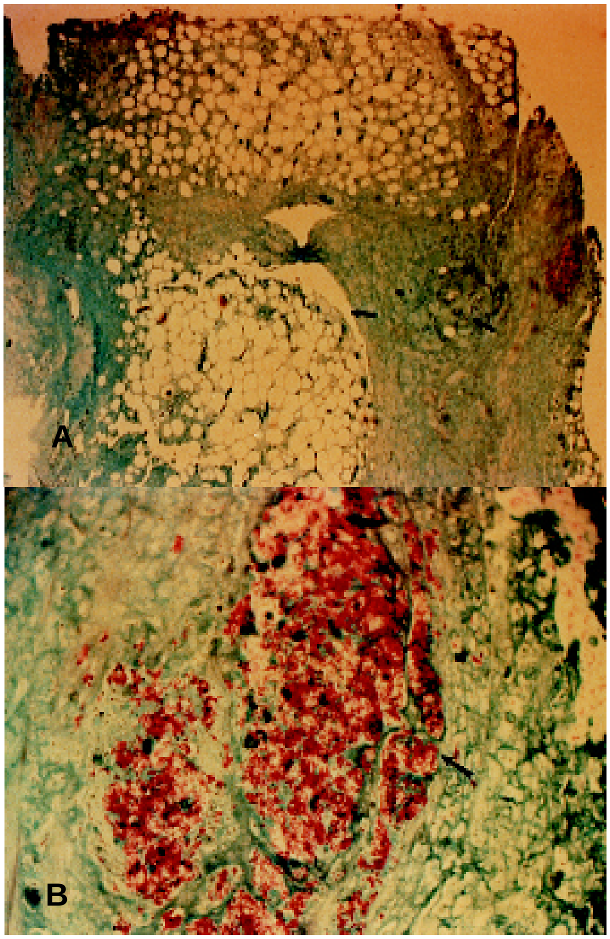

Figure 1. Early- (A) and late-stage (B) disease histopathologic sections of the dermis stained for acid-fast bacilli (AFB) from a patient with a *Mycobacterium ulcerans* infection. In A, arrows indicate necrosis of adipose tissue distant from the location of AFB, and in B, the arrow indicates predominance of extracellular bacilli and microcolonies. Patients' samples were obtained from the study conducted in Côte d'Ivoire (12).
(Photos courtesy of National Center for Infectious Diseases, CDC, Atlanta, Georgia.)

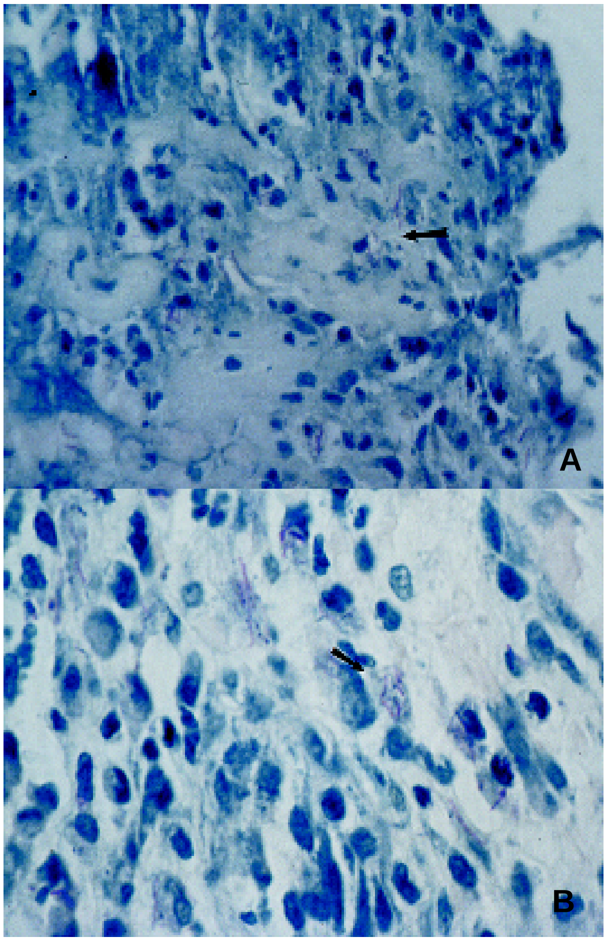

Figure 2 A and B. Active disease histopathologic sections of soft tissue stained for acid-fast bacilli from a patient with a *Mycobacterium marinum* infection. In A, the arrow indicates localized necrosis, and in B, the arrow indicates predominance of intracellular bacilli.
(Slide courtesy of Arthur B. Abt and Leslie Parent, Penn State Geisinger Health System and Pennsylvania State University College of Medicine, Hershey Medical Center, Hershey, Pennsylvania.)

spread is noted, with sporotrichoid lesions and lymphadenitis. These lesions may result in scarring but are less extensive than those caused by *M. ulcerans*; deformity is unusual. Disease in patients with AIDS has been reported (25), and dissemination may occur in the immunosuppressed (26,27).

The bacilli are located throughout the necrotic lesions (Figure 2A), with many bacilli observed as singular rods within cells and vacuoles (Figure 2B). These lesions swell progressively as the infection ensues until nodules are formed. Tissue necrosis usually occurs at small sites within these nodules and is observed only in close proximity to the AFB (Figure 2A). Unlike lesions of *M. ulcerans*, the histopathologic features of early *M. marinum* lesions are similar to those of the lesions observed in pulmonary tuberculosis (24). *M. marinum* lesions generally show nonspecific inflammation followed by granuloma formation (24). Very few AFB are observed in the lesions themselves; they are present as single or a few bacilli without microcolonies (Figure 2A, B); however, the organism can be cultured from the skin lesion.

### *M. haemophilum* Disease

*M. haemophilum* generally causes joint and cutaneous infections in immunocompromised patients and lymphadenitis and cutaneous lesions in healthy children (19,28). In addition, a recent report has described cutaneous lesions arising from infection with *M. haemophilum* in two healthy men with no other risk factors for disease (19). Lesions often begin as raised, violaceous nodules, most commonly on the extremities (19,28). In one report, onset of disease occurred approximately 16 months after the onset of AIDS (28). Nodules frequently become erythematous and ulcerated, and recurrence of ulcers may occur in patients in whom complete lesions were not excised (19).

Unlike *M. marinum* lesions, *M. haemophilum* lesions are not sporotrichoid and do not appear to localize to areas above the lymphoid tissues; they are more frequently found above joints, especially appearing around submandibular and cervical joints in infections in children. Mature lesions are often extremely painful, in contrast to mature lesions caused by *M. marinum* and *M. ulcerans*. The organism may also cause septic arthritis or respiratory disease and can be associated with systemic symptoms such as fevers and night sweats. Disseminated disease occurs in severely immunodeficient persons (20). In one report, 9 of 13 immunocompromised patients died, although death may have been due to conditions other than *M. haemophilum* infection (28).

*M. haemophilum*-infected skin shows minute necrotic foci in the deep dermis surrounded by granulocytes, lymphocytes, monocytes, fusiform cells, and a few giant cells of the Langerhans type (29). Large numbers of bacilli are generally observed both extracellularly and intracellularly as singular cells within these necrotic foci (Figure 3A, B). Histopathologic examinations also reveal poorly formed granulomas within the ulcerated skin lesions (Figure 3B). Similarly, AFB are observed inside and outside the cells and as microcolonies within these granulomas and in the surrounding tissue (Figure 3A, B).

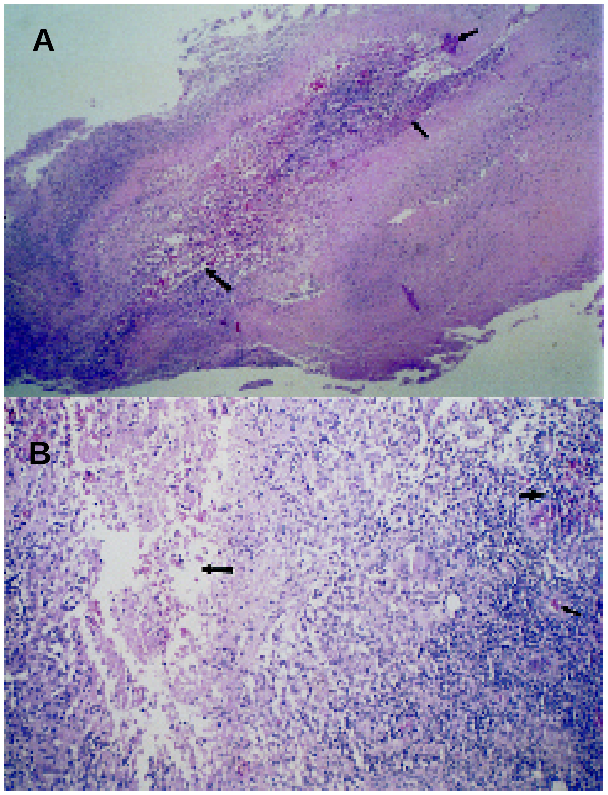

Figure 3 A and B. Active disease histopathologic sections of the epidermis stained for acid-fast bacilli from a patient infected with *Mycobacterium haemophilum*. In A and B, the arrows indicate localized necrosis and presence of intracellular and extracellular bacilli and microcolonies and the presence of loose granulomas.

(Slide courtesy of Michael A. Saubolle, Good Samaritan Regional Medical Center, Phoenix, Arizona, and Department of Medicine, University of Arizona School of Medicine, Tucson, Arizona.)

### Pathogenesis

One hallmark of most diseases caused by mycobacteria is the ability of the bacilli to grow within host cells. *M. haemophilum* and *M. marinum* grow prolifically within fibroblast, epithelial cells (Figures 2, 3) (30,31) and macrophages (29,32). In contrast, *M. ulcerans* primarily forms extracellular microcolonies within necrotic tissues, is rarely found within host cells, and disrupts macrophages and adipose cell monolayers in vitro in lieu of growing within these cells (33; our unpub. obs.). Rastogi et al. (34) showed that although *M. ulcerans* would infect and persist in murine macrophages after 4 days, no intracellular growth occurred. Others have demonstrated that culture filtrates from *M. ulcerans* suppressed phagocytosis of the bacilli and speculated that this in vitro suppression of phagocytosis is the reason that the bacilli are only rarely observed within host cells in human disease (33).

The necrosis in the skin lesions of all three mycobacterial infections suggests a secreted or somatic cytotoxin or other necrotic bacterial component.

### Buruli Ulcer

The pre-ulcerative and early ulcer stages of BU are characterized by a central zone of microcolonies of AFB surrounded by a larger zone of necrotic tissue with no evidence of host-derived inflammatory exudates that might contribute to cytotoxicity (35). Further, culture filtrates from *M. ulcerans* produce a cytotoxic effect on cultured fibroblasts (35). This material simulated clinical and histopathologic changes similar to those in BU when injected into guinea pigs (36). An initial analysis of the culture filtrates identified a high molecular weight phospholipoprotein-polysaccharide complex that retained the ability to produce a cytotoxic effect on cell monolayers (37). Others have ascribed the cytotoxic effect of *M. ulcerans* to a low molecular weight lipid (742 daltons) in the filtrates (38). Fractionation of the culture filtrates and observation of the effects of each fraction on cultured L929 fibroblast cells initially identified a lipid as the cytotoxic component. Further purification and analysis of this lipid did not induce cell death, but rather arrested the cellular growth (38).

Some suggest that the factors secreted by *M. ulcerans* may also possess immunosuppressive properties indirectly contributing to the destruction of human tissue (33). Others believe that the necrosis of tissue is due primarily to infarction, with no contribution from cytotoxic bacterial factors (23). Thus, the overall cytotoxic effects demonstrated by *M. ulcerans* in human disease may result from multiple factors. No study has addressed whether cytotoxic or immunosuppressive factors are released from *M. ulcerans* during the early, active, or late stages of infection; the mechanisms by which such factors might act on host tissues are also not known.

### *M. marinum* Disease

In *M. marinum* infection, bacilli are capable of invading and replicating within cultured macrophages and epithelial cells (31). Intracellular growth of *M. marinum* is limited at temperatures above 33°C, as the bacilli do not grow intracellularly at 37°C. In one study, temperature-dependent growth also correlated with cytotoxicity of macrophage monolayers, but no evidence of secreted toxins was noted from supernatants of these infected tissue cultures. The investigators suggested that intracellular growth and a faster growth rate at 33°C probably caused cytotoxicity (31).

*M. marinum* produces skin lesions in animal models without prior induced immunosuppression. Intravenous injection of *M. marinum* into normal mice caused skin lesions similar to those observed in humans, but no dissemination of the organism occurred (40). Dissemination of *M. marinum* was induced only when mice or leopard frogs (*Rana pipiens*) were subjected to conditions that lower the immune response, such as lower body temperatures or treatment with hydrocortisone (40,41). A strain adapted to an optimal growth temperature of 37°C in broth culture produced immediate disseminated disease when injected into the foot pads or tail veins of normal mice (40), demonstrating that once temperature restriction was removed as a barrier to growth, the bacilli quickly disseminated regardless of immune status of the mice.

### *M. haemophilum* Disease

A putative cytotoxin from *M. haemophilum* has not been reported, even though the presence of such a toxin is suggested by histopathologic examinations of infected tissues and in vitro tissue culture studies (30). In particular, an epithelial cell tissue culture model demonstrated that *M. haemophilum* induced substantial cytotoxicity in epithelial cells at 33°C but not at 37°C, even though the bacilli grew extracellularly in coculture with epithelial cells at 37°C (30). Filtered supernatant from 33°C infected tissue cultures produced identical cytotoxic reaction when layered onto fresh monolayers. However, unlike from *M. ulcerans*, broth medium from bacterial culture was not cytotoxic. These studies suggest that a cytotoxin may be produced only upon infection of epithelial cells growing at 33°C. Intracellular growth occurred only during infections at 33°C, even though electron microscopy showed that the bacilli were capable of invading these cells at 37°C (30). This temperature-specific cytotoxicity and intracellular growth mimic the clinical signs of infection in humans. The bacilli grow in cooler, superficial regions of the body where primary tissue destruction occurs; subsequently, the bacilli may spread to deeper, warmer tissues of the host, where little tissue disruption is observed and granulomas form around the infected areas (19).

Systemic symptoms have been reported but were not likely caused by a cytotoxin released by the bacteria (19). Animal studies suggest that immunosuppression leads to disseminated *M. haemophilum* disease. The development of skin lesions and dissemination by *M. haemophilum* do not occur when the bacilli are injected intravenously into healthy mice. However, upon inoculation with *M. haemophilum*, mice treated with steroids to induce immunosuppression develop skin lesions and disseminated disease similar to human disease (39).

### Epidemiology

### Buruli Ulcer

The reservoir of *M. ulcerans* is unknown. The organism has only been recovered from lesions of humans or, in one case, a koala (42), and there has been only one report of person-to-person spread (9). Thus, environmental exposure, either by direct inoculation or an insect vector, is the likely route of infection. Epidemiologic studies suggest that proximity to water sources, such as freshwater lakes or rivers, predisposes to disease, but specific contact with water that might lead to transmission of the bacteria has not been identified. In fact, one study showed that BU was more common during the dry season (43). Cultures of water near BU-endemic areas have not yielded *M. ulcerans* (9,12,43,44), though testing of water samples by polymerase chain reaction found *M. ulcerans* DNA (44,45). Soil also has been considered a possible reservoir, but the organism has not been isolated from soil samples.

BU is primarily a disease of children, with the highest rates found in children ages 2 to 14 years (12). Boys and girls are equally affected. In some areas, women also have an elevated risk of BU (12). No data are available on disease rates by race, but the disease has been reported in all racial groups. Although reported in persons with HIV (46,47), no predilection for BU has been noted in HIV-infected persons or other immunodeficient patients, despite the substantial rates of HIV in many BU-endemic areas (9,48).

### *M. marinum* Disease

Water is the source of infection by *M. marinum* in humans (24). Recovered from unchlorinated swimming pools and salt and fresh water aquariums associated with cases of disease, the organism is also a common pathogen of fish; however, water is likely the reservoir, and fish are a susceptible host. The organism may be transmitted through minimal trauma or abrasions to the skin. Person-to-person spread has not been well documented, and no geographic localization of the disease has been noted (41).

The mean age of persons with *M. marinum* disease is 35 to 42 years (17,24), although cases have been reported in all age groups. Caucasians, men, and urban residents are at highest risk. Like BU, the disease is not associated with immunosuppression and is rare in AIDS patients, even though cases are common in industrialized countries where AIDS is prevalent (25-27).

### *M. haemophilum* Disease

Unlike *M. ulcerans* and *M. marinum*, most *M. haemophilum* infections occur in immunocompromised patients (20). There is scant evidence of person-to-person spread of *M. haemophilum*, similar to that observed for *M. ulcerans* and *M. marinum* infections. *M. haemophilum* disease also appears to be acquired from the environment, but the reservoir is unknown; the organism has been isolated only from humans with disease (20,49). A case-control study that included cases apparently acquired through nosocomial transmission did not find common epidemiologic elements (28). Another study considered the role of aerosolized pentamidine in the spread of *M. haemophilum*. In this study, six patients at a cancer center contracted *M. haemophilum* disease after prophylactic therapy with aerosolized pentamidine, with two of these patients having *M. haemophilum* pneumonia. The role of this therapeutic treatment in exposure could not be clarified either, as numerous patients receiving this therapy did not contract *M. haemophilum,* nor was the organism recovered from the reconstituted pentamidine or water used before and after nebulization (19). Because cases have occurred in persons residing near the ocean or large lakes, water has been suggested as a possible reservoir (20,50). This conjecture, however, does not explain the increase in the number of cases observed in the southwestern United States, particularly Arizona (19).

The median age of persons with *M. haemophilum* disease is 31 years, and 80% were immunosuppressed (20). Two thirds of cases were in men, and most were in Caucasians.

### Factors Relevant to the Mode of Transmission

Mycobacteria are widespread in the environment, particularly in aquatic reservoirs. In one survey, more than 67% of water specimens collected from natural, treated, and animal-contact sources contained mycobacteria, including *M. marinum* (50). Mycobacteria also are commonly found in soil. Wolinsky and Rynearson (51) identified at least one *Mycobacterium* species from 86% of the samples they collected from several locations. *M. haemophilum* was not recovered from any samples in this survey, probably because the culture methods used were not suitable for the growth of this *Mycobacterium*.

### *M. ulcerans*

*M. ulcerans* grows slowly at all temperatures between 25°C and 37°C, although greater proliferation is observed during growth at temperatures between 30°C and 33°C. No colony variants have been reported, and the bacterium apparently has a shorter doubling time in a medium enriched with fatty acids, a phenomenon consistent with bacilli in lipid-rich areas surrounding sites of infection (6,9).

Singular lesions involving areas of the body most susceptible to trauma (i.e., upper and lower extremities) are frequently observed, and direct inoculation is the most plausible mode of transmission for *M. ulcerans* infection. Numerous case histories document skin trauma and abrasions preceding the onset of BU (9,12). Others propose that an insect vector may transmit *M. ulcerans* (54), although supporting evidence for this hypothesis has not been reported.

*M. ulcerans* infections may be linked to environmental disturbances (9,12,23,43). The first reported cases of *M. ulcerans* infection occurred 2 or 3 years after severe regional flooding and continued intermittently until 1950 (7). In 1978, there again was severe flooding in this area, and again approximately 2 years later, infections occurred, first in koalas and later in humans. Cases of BU were also observed on the east side of the Victoria Nile in Uganda between 1962 and 64 (43); this outbreak was also likely associated with severe flooding in the region caused by heavy rains. As in the outbreak observed in Australia, 2 or 3 years elapsed between the flooding in Uganda and the first cases in the region. In Togo, infection in children was related to seasonal flooding of rivers in proximity to the local village (55). Cases were reported in one area in Liberia after swamp rice was introduced to replace upland rice. This introduction was associated with the construction of dams on a major river and the artificial extension of wetlands (56). In addition, recent cases described in Côte d'Ivoire occurred primarily in association with farming activities near the main river (12). Therefore, a common feature of *M. ulcerans* disease is that infected persons often reside near swampy areas, river valleys, or lakes and coastal areas.

After a flood or some other environmental disturbance, mycobacteria may be washed from their normal habitat into draining rivers or lakes. Given favorable circumstances, such as relative stream stagnation, temperatures of 27°C to 33°C, low salinity, low pH, and the presence of adequate nutrients, survival and growth of the organism may be enhanced. With the temperatures that are reached in the tropics, moderate temperatures in the surface water and in moist silt beside lakes may be sufficient to sustain the growth of *M. ulcerans* during the daytime for most of the year (57). Moreover, *M. ulcerans* could be dispersed from a water environment in a fashion analogous to that documented for aquatic *M. avium*, where droplet aerosolization of the organism may result in infection (50,58), also suspected in recent BU cases in Bairnsdale, Australia. Most of the patients infected with *M. ulcerans* resided in a small region near one of the lakes but showed no history of direct contact with the water (57). This type of airborne dispersal would also explain the acquisition of BU in tree-living koalas that may be exposed to contaminated aerosols generated in the adjacent lake system (59). Additionally, airborne dispersal might explain the prevalence of disease on a geographic continuum in all countries bordering the Gulf of Guinea in West Africa (6). Finally, spray aerosolization of *M. ulcerans* in recycled sewage water used to irrigate a golf course has been proposed as the route of infection for another series of cases in Australia (13).

### *M. marinum*

Unlike that of *M. ulcerans* and *M. haemophilum*, the etiology of *M. marinum* disease is well known. Of the three, *M. marinum* has the clearest association with water as the source of the infection (60). In 1926, *M. marinum* was first isolated and identified as the cause of saltwater aquarium fish deaths (61). Tuberculoid skin lesions in users of a swimming pool in 1939 and 1954 and granulomatous mycobacterial disease in freshwater fish in 1942 also were ultimately attributed to *M. marinum* (62). Since then, a variety of skin infections due to this organism have been observed around the world, and names such as “mariner’s TB,” “aquarium granuloma,” and “swimming pool granuloma” have been coined to describe the disease as well as the source of the infection. However, when swimming pools are properly chlorinated, this association has all but disappeared (60). Nevertheless, virtually any water source and water-related activity is a potential risk, including tending aquariums (62), fishing (63), skin diving (62), and a number of other water-related activities (64).

### *M. haemophilum*

*M. haemophilum* is fastidious, grows slowly, requires supplemental iron, and has a lower incubation temperature for growth than most other mycobacteria. This organism is usually grown on chocolate agar, on egg-based media, or on Middlebrook media containing 15 µg/mL to 25 µg/mL ferric ammonium citrate, 0.4% hemoglobin, 60 µmol/L hemin, or on X-factor (52). The temperature growth range is 25°C to 35°C, but the optimal incubation temperature is 32°C (52). Because of these requirements, *M. haemophilum* cannot be isolated by the standard techniques used in clinical laboratories, and its fastidiousness may account for the lack of isolation from environmental sources and patient specimens.

The ecology of *M. haemophilum* is poorly understood, and the reservoir and modes of transmission still need to be elucidated. However, one study describing *M. haemophilum* isolates from different patients in the same hospital that had identical fingerprint patterns (53) supports the possibility that the patients were exposed to a common source, such as water. Additional evidence that *M. haemophilum* grows over a wide pH range (49) and can use chelated iron (52), characteristics common to other aquatic bacteria, suggests an environmental niche. This organism survives in cold water (our unpub. obs.) and is resistant to chlorine (50). Several researchers have suggested using frogs as models for the study of *M. haemophilum* systemic disease, as they (or other amphibians) have cooler body temperatures and thus could be environmental sources for this organism (15).

Possible modes of transmission for infection with *M. haemophilum* include inhalation, ingestion, and skin inoculation. Since most infections occur on the skin, direct inoculation may be most likely. However, patients were no more likely than healthy persons from the area to recall injuries or skin conditions before the onset of symptoms (19), and patients generally did not report cutaneous injuries before illness (19). The isolation of *M. haemophilum* from sputum and lung tissue suggests the possibility of respiratory transmission; however, respiratory therapies, exposure to irritants, or previous respiratory infections have rarely been associated with infection (19). Interestingly, an early animal model demonstrated that *M. haemophilum* can spread to the skin through dissemination in the blood after intravenous inoculation, suggesting bloodborne transmission (39). This study was performed on prednisolone-treated mice challenged intravenously with *M. haemophilum;* skin lesions (predominantly on the cooler regions of the body) developed in 12 of 30 mice.

This collective evidence illustrates a defined reservoir and mode of transmission for *M. marinum* infections, and, although similar evidence is described for *M. ulcerans* and *M. haemophilum*, the precise mode of transmission of these infections remains undefined.

### Conclusions

We have discussed the emergence of three skin diseases and presented the pathophysiologic, epidemiologic, and environmental characteristics that may contribute to their emergence.

Disease caused by *M. ulcerans* and *M. marinum* is primarily in immunocompetent persons, while the emergence of *M. haemophilum* disease is primarily in immunosuppressed persons. All three mycobacteria are thought to be acquired by inoculation during contact with contaminated water, suggesting that changes in the environment may be contributing to their emergence.

Histologic studies demonstrate that *M. ulcerans* has an apparent association with adipose tissue, is not observed within host cells in vivo, and does not grow intracellularly within cultured macrophages. In contrast, *M. marinum* and *M. haemophilum* grow intracellularly within cultured macrophages and epithelial cells and have apparent associations with epithelial tissues in vivo, suggesting that different cellular tropisms occur between these species after the dermis is initially infected. None of these organisms readily cause disseminated disease in immunocompetent humans, and this restriction may be related to the optimal growth temperature of these mycobacteria (temperatures similar to that of the human dermis), although all three can grow at warmer temperatures, albeit to a lesser extent in vitro. *M. marinum* colony variants that grow best at 37°C in vitro overcome this apparent temperature restriction in vivo and disseminate in animals, further demonstrating that while temperature-restricted growth likely limits the spread of disease, this restriction can be surmounted by acquired factors. *M. haemophilum* can become a disseminated disease in immunosuppressed persons, suggesting that the organism can also overcome temperature restrictions in a favorable environment.

The common source of infection for these three mycobacteria is likely water (it is the definitive source for *M. marinum)*. As with the source of infection, the mode of transmission has been clearly defined as inoculation after skin abrasion for *M. marinum*, while no clear association with skin abrasion has been demonstrated for infection with *M. ulcerans* or *M. haemophilum* in humans. Experimental inoculation of all three organisms into the skin of animals produces pathologic effects similar to those in humans. However, epidemiologic studies on *M. ulcerans* and *M. haemophilum* and disease and laboratory experiments with these two organisms in animals suggest different modes of transmission than *M. marinum*, including possible aerosol transmission with subsequent hematogenous spread to the dermis.

Finally, comparisons of the pathogenic and histopathologic characteristics of these three diseases suggest differences in the levels of virulence, mechanisms of pathogenesis, and modes of transmission. As we learn more about the ecology, epidemiology, and pathogenesis of these unique mycobacteria through improvements in culture techniques, diagnostic tests, and continued laboratory research, we may be able to identify contaminated environments contributing to the source of these infections and elucidate the mode of transmission. Such information is essential if we are to develop strategies to prevent the further emergence of these diseases.

### Acknowledgments

The authors thank Barbara J. Marston and Jordan Tappero for their advice and consultation regarding BU, Leslie Parent for histology slides of *M. marinum* infection and her consultation, Michael A. Saubolle for histology slides of *M. haemophilum* infection and his consultation, Bryan R. Davis for his consultation regarding disseminated *M. haemophilum* infections, Laura Povinelli for critical review of the PHLIS data, and Jennifer Ekmark and Ellen Spotts for critical review of this submission.

Dr. Dobos is a postdoctoral fellow working on the molecular pathogenesis and serodiagnosis of *Mycobacterium ulcerans* infection at The Emory University School of Medicine and CDC. She received her graduate training at Colorado State University, where she was the first to describe the glycosylation of a *Mycobacterium tuberculosis* protein.

### References

1. Murray CJ, Styblo K, Rouillon A. Tuberculosis in developing countries: burden, intervention and cost. Bulletin of the International Union of Tuberculosis and Lung Disease 1990;65:6-24.
2. Raviglione MC, Narain JP, Kochi A. HIV-associated tuberculosis in developing countries: clinical features, diagnosis, and treatment. Bull World Health Organ 1992;70:515.
3. Falkinham JO. Epidemiology of infection by nontuberculosis mycobacteria. Clin Microbiol Rev 1996;9:177-215.
4. Horsburgh CR Jr. *Mycobacterium avium* complex infection in the acquired immunodeficiency syndrome. N Engl J Med 1996;324:1332-8.
5. Horsburgh CR Jr. Epidemiology of diseases caused by nontuberculosis mycobacteria. Semin Respir Infect 1996;11:244-54.
6. World Health Organization targets untreatable ulcer: report from the first international conference on Buruli ulcer control and research. Yamousoukro (Côte d'Ivoire): Inter Press Service; 1998 Jul 31.

7. MacCallum P, Tolhurst JC, Buckle G, Sissons HA. A new mycobacterial infection in man. Journal of Pathologic Bacteriology 1948;60:93-122.
8. Dodge OG, Lunn HF. Buruli ulcer: a mycobacterial skin ulcer in a Uganda child. Journal of Tropical Medicine and Hygiene 1962;65:139-42.
9. Horsburgh CR Jr, Meyers WM. Buruli ulcer. In: Horsburgh CR Jr, Nelson AM, editors. Pathology of emerging infections. Washington: American Society for Microbiology Press; 1997. p. 119-26.
10. Dawson JF, Allen GE. Ulcer due to *Mycobacterium ulcerans* in Northern Ireland. Clin Exp Dermatol 1985;10:572-6.
11. Kozin SH, Bishop AT. Atypical mycobacterial infections of the upper extremity. J Hand Surg 1994;19:480-7.
12. Marston BJ, Diallo MO, Horsburgh CR Jr, Diomande I, Saki MZ, Kanga JM, et al. Emergence of Buruli ulcer disease in the Daloa region of Côte d'Ivoire. Am J Trop Med Hyg 1995;52:219-24.
13. Johnson PDR, Veitch MG, Leslie DE, Flood PE, Hayman JA. The emergence of *Mycobacterium ulcerans* infection near Melbourne. Med J Aust 1996;164:76-8.
14. Feldman RA, Hershfield E. Mycobacterial skin infection by an unidentified species. A report of 29 patients. Ann Intern Med 1974;80:445-52.
15. Sompolinsky D, Lagziel A, Naveh D, Yankilevitz T. *Mycobacterium haemophilum* sp. nov, a new pathogen of humans. Int J Syst Bacteriol 1978;28:67-75.
16. Gouby A, Branger B, Oules R, Ramuz M. Two cases of *Mycobacterium haemophilum* infection in a renal dialysis unit. J Med Microbiol 1988;25:299-300.
17. O'Brien RJ, Geiter LJ, Snider DE. The epidemiology of nontuberculous mycobacterial diseases in the United States. American Review of Respiratory Disease 1987;135:1007-14.
18. Bean NH, Martin SM, Bradford H Jr. PHLIS: an electronic system for reporting public health data from remote sites. Public Health Briefs 1992;82:1273-6.
19. Saubolle MA, Kiehn TE, White MH, Rudinsky MF, Armstrong D. *Mycobacterium haemophilum*: microbiology and expanding clinical and geographic spectra of disease in humans. Clin Microbiol Rev 1996;9:435-47.
20. Kiehn TE, White M. *Mycobacterium haemophilum*: an emerging pathogen. Eur J Clin Microbiol Infect Dis 1994;13:925-31.
21. Armstrong KL, James RW, Dawson DJ, Francis PW, Masters B. *Mycobacterium haemophilum* causing perihilar or cervical lymphadenitis in healthy children. J Pediatr 1992;121:202-5.
22. Hayman J. Clinical features of *Mycobacterium ulcerans* infection. Australian Journal of Dermatology 1985;26:67-73.
23. Hayman J. Out of Africa: observations on the histopathology of *Mycobacterium ulcerans* infection. J Clin Pathol 1993;46:5-9.
24. Huminer D, Pitlik SD, Block C, Kaufman L, Amit S, Rosenfeld JB. Aquarium-borne *Mycobacterium marinum* skin infection. Report of a case and review of the literature. Arch Dermatol 1986;122:698-703.
25. Hanau LH, Leaf A, Soeiro R, Weiss LM, Pollack SS. *Mycobacterium marinum* infection in a patient with the acquired immunodeficiency syndrome. Cutis 1994;54:103-5.
26. Tchornobay AM, Claudy A, Perrot JL, Levigne M, Denis M. Fatal disseminated *mycobacterium marinum* infection. International Journal of Dermatology 1992;31:286-7.
27. Parent LJ, Salam MM, Appelbaum PC, Dossett JH. Disseminated *Mycobacterium marinum* infection and bacteremia in a child with severe combined immunodeficiency. Clin Infect Dis 1995;21:1325-7.
28. Straus WL, Ostroff SM, Jernigan DB, Kiehn TE, Sordillo EM, Armstrong D, et al. Clinical and epidemiologic characteristics of *Mycobacterium haemophilum*, an emerging pathogen in immunocompromised patients. Ann Intern Med 1994;120:118-25.
29. Sompolinsky D, Lagziel A, Rosenberg I. Further studies of a new pathogenic mycobacterium (*M. haemophilum* sp. nov.). Can J Microbiol 1979;25:217-26.
30. Fischer LJ, Quinn FD, White EH, King CH. Intracellular growth and cytotoxicity of *Mycobacterium haemophilum* in a human epithelial cell line (Hec-1-B). Infect Immun 1996;64:269-76.
31. Ramakrishnan L, Falkow S. *Mycobacterium marinum* persists in cultured mammalian cells in a temperature-restricted fashion. Infect Immun 1994;62:3222-9.
32. Mor N. Multiplication of *Mycobacterium marinum* within phagolysosomes of murine macrophages. Infec Immun 1985;48:850-2.
33. Pimsler M, Sponsler TA, Meyers WM. Immunosuppressive properties of the soluble toxin from *Mycobacterium ulcerans*. J Infect Dis 1988;157:577-80.
34. Rastogi N, Blom-Potar MC, David HL. Comparative intracellular growth of difficult-to-grow and other mycobacteria in a macrophage cell line. Acta Leprol 1989;7:156-9.
35. Read RG, Heggie CM, Meyers WM, Connor DH. Cytotoxic activity of *Mycobacterium ulcerans*. Infect Immun 1974;9:1114-22.
36. Krieg RE, Hockmeyer WT, Connor DH. Toxin of *Mycobacterium ulcerans*: production and effects in guinea pig skin. Arch Dermatol 1974;110:783-8.
37. Hockmeyer WT, Krieg RE, Reich M, Johnson RD. Further characterization of *Mycobacterium ulcerans* toxin. Infect Immun 1978;21:124-8.
38. George KM, Barker LP, Welty DM, Small PLC. Partial purification and characterization of biological effects of a lipid toxin produced by *M. ulcerans*. Infect Immun 1998;66:587-93.
39. Abbott MR, Smith DD. The pathogenic effects of *Mycobacterium haemophilum* in immunosuppressed albino mice. J Med Microbiol 1980;13:535-40.
40. Clark HF, Shepard CC. Effect of environmental temperatures on infection with *Mycobacterium marinum* (balnei) of mice and a number of poikilothermic species. J Bacteriol 1986;86:1057-69.
41. Ramakrishnan L, Valdivia RH, Mckerrow JH, Falkow S. *Mycobacterium marinum* causes both long-term subclinical infection and acute disease in the leopard frog (*Rana pipiens*). Infect Immun 1997;65:767-73.
42. Mitchell PJ, Jerrett IV, Slee KJ. Skin ulcers caused by *Mycobacterium ulcerans* in koalas near Bairnsdale, Australia. Pathology 1984;16:256-60.
43. Barker DJP. The distribution of Buruli disease in Uganda. Trans R Soc Trop Med Hyg 1972;66:867-74.

44. Ross BC, Johnson PD, Oppedisano F, Marino L, Sievers A, Stinear T, et al. Detection of *Mycobacterium ulcerans* in environmental samples during an outbreak of ulcerative disease. Appl Environ Microbiol 1997;63:4135-8.
45. Roberts B, Hirst R. Immunomagnetic separation and PCR for detection of *Mycobacterium ulcerans*. J Clin Microbiol 1997;35:2709-11.
46. Ameh EA, Dogo PM, Ahmed A, Maitama HY, Esangbedo AE, Nmadu PT. *Mycobacterium ulcerans* skin infection in a patient with HIV infection: is this incidental? Trop Doct 1997;27:59.
47. Delaporte E, Savage C, Alfandari S, Piette F, Leclerc H, Bergoend H. Buruli ulcer in a Zairian woman with HIV infection. Ann Dermatol Venereol 1994;121:557-60.
48. Allen S. Buruli ulcer and HIV infection. Int J Dermatol 1992;31:744-5.
49. Portaels F, Dawson DJ, Larsson L, Rigouts L. Biochemical properties and fatty acid composition of *Mycobacterium haemophilum*: study of 16 isolates from Australian patients. J Clin Microbiol 1993;31:26-30.
50. Collins CH, Grange JM, Yates MD. A review: mycobacteria in water. Journal of Applied Bacteriology 1984;57:193-211.
51. Wolinsky E, Rynearson TK. Mycobacteria in soil and their relation to disease-associated strains. American Review of Respiratory Disease 1968;97:1032-7.
52. Dawson DJ, Jennis F. Mycobacteria with a growth requirement for ferric ammonium citrate, identified as *Mycobacterium haemophilum*. J Clin Microbiol 1980;11:190-2.
53. Kikuchi K, Bernard EM, Kiehn TE, Armstrong D, Riley LW. Restriction fragment length polymorphism analysis of clinical isolates of *Mycobacterium haemophilum*. J Clin Microbiol 1994;32:1763-7.
54. Radford AJ. *Mycobacterium ulcerans* in Australia. Aust N Z J Med 1975;5:162-9.
55. Meyers WM, Tignokpa N, Priuli GB, Portaels F. *Mycobacterium ulcerans* infection (Buruli ulcer): first reported patients from Togo. Br J Dermatol 1996;134:1116-21.
56. Monson MH, Gibson DW, Connor DH, Kappes R, Heinz HA. *Mycobacterium ulcerans* in Liberia: a clinicopathologic study of 6 patients with Buruli ulcer. Acta Trop 1984;41:165-72.
57. Hayman J. Postulated epidemiology of *Mycobacterium ulcerans* infection. Int J Epidemiol 1991;20:1093-8.
58. Wendt SL, George KL, Parker BC, Gruft H, Falkinham JO. Epidemiology of nontuberculous mycobacteria. III. Isolation of potentially pathogenic mycobacteria in aerosols. American Review of Respiratory Disease 1980;122:259-63.
59. McOrist S, Jerrett IV, Anderson M, Hayman J. Cutaneous and respiratory tract infection with *Mycobacterium ulcerans* in two koalas (*Phascolarctos cinereus*). J Wildl Dis 1985;21:171-3.
60. Gluckman SJ. *Mycobacterium marinum*. Clin Dermatol 1995;13:273-6.
61. Aronson JD. Spontaneous tuberculosis in salt water fish. Infectious Disease 1926;39:315-20.
62. Collins CH, Grange JM, Noble WC, Yates MD. *Mycobacterium marinum* infections in man. Journal of Hygiene (Cambodia) 1985;94:135-49.
63. McClain EH. Case study: mariner's TB. American Association of Occupational Health Nurses Journal 1989;37:329-32.
64. Flowers DJ. Human infection due to *Mycobacterium marinum* after a dolphin bite. Journal of Clinical Pediatrics 1970;23:475-7.
